# EANM enabling guide: how to improve the accessibility of clinical dosimetry

**DOI:** 10.1007/s00259-023-06226-z

**Published:** 2023-04-22

**Authors:** Jonathan Gear, Caroline Stokke, Christelle Terwinghe, Silvano Gnesin, Mattias Sandström, Johannes Tran-Gia, Marta Cremonesi, Francesco Cicone, Fredrik Verburg, Roland Hustinx, Luca Giovanella, Ken Herrmann, Pablo Minguez Gabiña

**Affiliations:** 1 Joint Department of Physics, Royal Marsden NHSFT & Institute of Cancer Research, Sutton, UK; 2 Division of Radiology and Nuclear Medicine, Oslo University Hospital, Oslo, Norway; 3 Department of Physics, University of Oslo, Oslo, Norway; 4 Department of Nuclear Medicine, Universitair Ziekenhuis Leuven, Louvain, Belgium; 5 Institute of Radiation Physics, Lausanne University Hospital, University of Lausanne, Lausanne, Switzerland; 6 Section of Nuclear Medicine and PET, Department of Surgical Sciences, Uppsala University, Uppsala, Sweden; 7 Sweden & Section of Medical Physics, Department of Immunology, Genetics and Pathology, Uppsala University, 751 85 Uppsala, Sweden; 8 Department of Nuclear Medicine, University Hospital Würzburg, Oberdürrbacher Str. 6, 97080 Würzburg, Germany; 9 Radiation Research Unit, Department of Medical Imaging and Radiation Sciences, Istituto Europeo Di Oncologia, IRCCS, Milan, Italy; 10 Department of Experimental and Clinical Medicine, Neuroscience Research Centre, PET/RM Unit, “Magna Graecia” University of Catanzaro, Catanzaro, Italy; 11 Nuclear Medicine Unit, University Hospital “Mater Domini, Catanzaro, Italy; 12 Department of Radiology and Nuclear Medicine, Erasmus Medical Center, Rotterdam, the Netherlands; 13 Division of Nuclear Medicine and Oncological Imaging, University Hospital of Liège, Liège, Belgium; 14 GIGA-CRC in Vivo Imaging, University of Liège, Liège, Belgium; 15 Clinic for Nuclear Medicine and Molecular Imaging, Imaging Institute of Southern Switzerland, Ente Ospedaliero Cantonale, Bellinzona, Switzerland; 16 Department of Nuclear Medicine, University of Duisburg-Essen, Duisburg, Germany; 17 German Cancer Consortium (DKTK)-University Hospital Essen, Essen, Germany; 18 Department of Medical Physics and Radiation Protection, Gurutzeta-Cruces University Hospital/Biocruces Health Research Institute, Barakaldo, Spain; 19 Department of Applied Physics, Faculty of Engineering, UPV/EHU, Bilbao, Spain

**Keywords:** Dosimetry, Molecular radiotherapy, SPECT, Nuclear medicine, Quantitative imaging, Optimisation

## Abstract

Dosimetry can be a useful tool for personalization of molecular radiotherapy (MRT) procedures, enabling the continuous development of theranostic concepts. However, the additional resource requirements are often seen as a barrier to implementation. This guide discusses the requirements for dosimetry and demonstrates how a dosimetry regimen can be tailored to the available facilities of a centre. The aim is to help centres wishing to initiate a dosimetry service but may not have the experience or resources of some of the more established therapy and dosimetry centres. The multidisciplinary approach and different personnel requirements are discussed and key equipment reviewed example protocols demonstrating these factors are given in the supplementary material for the main therapies carried out in nuclear medicine, including [^131^I]-NaI for benign thyroid disorders, [^177^Lu]-DOTATATE and ^131^I-mIBG for neuroendocrine tumours and [^90^Y]-microspheres for unresectable hepatic carcinoma.

**Supplementary Information:**

The online version contains supplementary material available at 10.1007/s00259-023-06226-z.

## Introduction

Since the early introductions of radiopharmaceuticals for therapy, there has been continued interest in optimisation and personalisation, to determine the ideal treatment activities and regimens. Of the optimisation strategies developed, dosimetry approaches, such as those adopted by external beam radiation therapy (EBRT) or brachytherapy, arguably have the strongest scientific grounding with the treatment mechanism shown to be that of radiation induced cell kill.

In molecular radiotherapy (MRT), some treatment planning procedures have been reported in specific applications [1–5] and dose–effect relationships in several therapy procedures have been highlighted [6–8]. Studies have also exposed the relevant improvements reached by dosimetry-based approaches in terms of progression-free survival and overall survival [7–13]. Thus, from a clinical perspective, dosimetry could offer a valuable tool that can assist with treatment individualisation. However, unlike EBRT and brachytherapy, in MRT there is in general still a shortage of agreed absorbed dose thresholds for lesions or absorbed dose constraints for organs at risk (OARs) that could be prescribed. Well-designed studies aimed to provide robust dosimetry and response data, and prospective trials to confirm the findings, are therefore required to fully optimise therapies based on absorbed dose treatment planning.

In cases where dosimetry is not directly employed to individualise a therapy, there is still scope to use it to verify treatment delivery. Comparison of absorbed doses to that of population data can be used for evaluating likely response or toxicity. While this is of interest for all therapies, it may be particularly useful to inform a therapy with unusual clinical indications or where treatment outcome or toxicity is of particular concern. In such cases, a patient could be selected for increased monitoring or observation. Alternatively, it may be possible that additional cycles are stopped early, potentially saving the health authority the expense of a costly treatment and allowing the patient to move quickly to a more appropriate treatment strategy. This prospect for clinical and economic benefit, must be weighed up against the additional cost of the dosimetry and requires adequate dosimetry data available with which to compare and define a “normal range”.

Evidently for the widespread clinical benefit of dosimetry to be fully implemented, commitment from clinical centres to acquire and collate dosimetry data is required. Without first gathering such data, population dose distributions cannot be derived, nor “normal” ranges defined. Equally absorbed dose constraints and toxicity thresholds cannot be evaluated to inform the design of the necessary randomised controlled trials with which to definitively demonstrate improved efficacy.

In 2020, the EANM noted that interpretation of EC Directive 2013/59/Euratom, laying down basic safety standards (BSS) for protection against the dangers arising from exposure to ionising radiation [14] into practical application was still lacking across Europe. The EANM position statement proposed three different classes of treatment verification and optimisation [15] inspired by the indication of levels in prescribing, recording and reporting of absorbed doses after radiotherapy defined by the International Commission on Radiation Units and Measurements (ICRU) and later defined for radiopharmaceuticals in ICRU report 96 [16]. Recently, a joint EANM, SNMMI and IAEA enabling guide on how to set-up a theranostics centre was released [17, 18] advising centres on important considerations for delivering these therapies. With the ever-increasing variety of therapies, there is also a wide range of dosimetry methodologies availablethe integration of such approaches into a clinical service can be daunting. The EANM have further conducted a survey evaluating the potential time and personnel resources typically being dedicated to different aspects of dosimetry for MRT [19]. These data provide a useful perspective for centres to understand the practicality of the resource requirement for MRT dosimetry and explore methods of reducing that burden where possible.

In this document, we discuss the requirements for introducing dosimetry as part of the theranostic procedure and argue how a dosimetry regimen can be tailored to the available resources of a centre depending on the needs of the department, national regulations and which of the dosimetry levels is being considered. The aim is to help centres wishing to initiate a dosimetry service but may not have the experience or resources of the more established therapy and dosimetry centres. In the Supplementary Material example, dosimetry regimens are presented for some common therapies. For each example, two possible, but different dosimetry strategies are provided. The advantages and disadvantages of each method are summarized. These methodologies have been highlighted to demonstrate the vast differences in methods and resources that could be applied. These methodologies are neither exhaustive nor exclusive, and an alternative or combination of each technique could also be applied.

## Making dosimetry accessible

Careful preparation is needed before a dosimetry service or study can commence. This was highlighted in the EANM resource survey [19] which considered three separate steps in implementing dosimetry: (1) protocol development, (2) preparatory work, and finally (3) the patient studies.

### Developing a dosimetry protocol

The EANM survey reported that the median time required to derive and develop a clinical dosimetry protocol was 4 days. Appropriately, this process requires input from different disciplines to ensure that the technical, clinical and scientific aspects of the protocol are met. The survey suggested that input from medical physics, NM technologists and the medical practitioner was common. The EANM has an established portfolio of both clinical and technical dosimetry guidelines produced by multidisciplinary teams, and often prepared in conjunction with other international organisations such as the IAEA, SNMMI and the MIRD committee. It remains the ambition of the EANM to continue supporting the community in the production of these guidelines and help in the formation of clear and appropriate operating procedures related to dosimetry. Protocol choice will depend on the therapy, and the requirements of department. Consideration should also be given as to the personal and medical conditions of the patient, and protocols adjusted as necessary. Even for centres which do not expect to deliver dosimetry-guided therapy, it is good practise to have such systems of work in place in case a clinical case presents where verification and more specialised treatment optimisation is required. Furthermore, dose-reporting to national regulators in instances of accidental or unexpected exposure is usually a legislative requirement that MRT centres must comply. In cases of unexpected early or late toxicity effects, dosimetry documentation may help identifying or exclude possible contributions or causes (e.g. specific/newly identified risk factors) related to certain patients or specific clinical characteristics.

### Initial preparations and system configurations

Prior to commencing a dosimetry study, it is often necessary to undertake some initial preparatory work such as system commissioning, configuration and testing. These are generally required to obtain baseline or system characteristics and ensure the developed protocol is suitable prior to first use. Methodologies for such studies are well documented in the appropriate guidelines. Provided that the mandated regular quality control assessments are fulfilled, the periodicity of the specific dosimetry tests can be low (yearly, twice per year or quarterly). The most time intensive tests indicated by the EANM survey were the imaging tests. Comparatively, resource requirements for these are similar to that required on PET/CT systems for trial accreditation. For dosimetry and therapeutic applications, the radionuclides used are of a considerably longer half-life than positron emitters and therefore coordination of phantom preparation is arguably much easier as more time can be given between source preparation and scanning. There is also the added advantage that multiple gamma cameras can be tested with the same phantom preparation, further reducing resource requirements. Results from multi-centre comparison exercises and clinical trials have also demonstrated consistent system characteristics across similar SPECT models potentially negating the need to establish these for every system, provided similar acquisition protocols are adopted [20, 21].

For non-imaging preparatory work including detector calibration, resources are considerably less arduous and can very often be performed on a daily or patient basis. For example, to determine a conversion factor between whole-body activity and dose-rate measurements, a “self-calibration” technique consisting of a quick measurement of a few minutes acquired immediately after administration (before any voiding), can be used [22]. Conversely for other radiation detection systems, such as gamma well counters or thyroid uptake probes, sensitivity should be measured at regular intervals. Rather than undertaking complex phantom preparation each time, the sensitivity can be checked initially with the therapeutic radionuclide and then regularly monitored with a long-lived sealed source, such as that used for daily quality assurance of a dose calibrator.

### Dosimetry acquisitions and calculations

The EANM position paper on Directive 2013/59 proposed three levels of dosimetry and the resource requirements for these levels can be tailored appropriately to suit the clinical indication, the intent of the dosimetry and the resources of the department. Thus, the first step is to decide the aim of the dosimetry. This will then influence the required output (e.g. organs of interest) and the appropriate dosimetry method for that therapy and centre. The accuracy of a dose estimate will inevitably decrease with protocol simplifications (as outlined in the supplementary examples). However, this may be acceptable in many clinical scenarios, and the dosimetric approach should be guided based on the clinical need and acceptable level of uncertainty in dose estimate.

Dosimetry using patient cohort-averaged dose data requires very little resourcing beyond collating the typical doses reported in the literature for the therapy in question. This information can be gathered when first developing the therapy protocol and is often readily available in the appropriate guidance documents. For most MRT procedures, a range or distribution of absorbed doses have been reported, providing valuable indication of the likeliness of potential under- or over-dosing in a population. For an individual, cohort-based absorbed doses to pathologic and limiting tissues can be estimated according to the activity administered with the treatment delivery confirmed through post therapy imaging.

A personalised dose assessment following a therapy is often associated with the need to acquire SPECT/CT studies at multiple time-points spanning many days. However, significant work has been undertaken to validate practical methods to reduce the burden to the patient and department [23]. For centres with reduced capacity when delivering therapies over multiple cycles, dosimetry could be performed at alternate cycles, or just on the initial cycle. Alternatively, when post therapy imaging is being performed as part of level 1 verification, it is often not a substantial effort to develop this into a quantitative image. A combination of the patient-specific quantitative measurement with population effective half-lives can, for some MRT procedure and organs, enable a population-based absorbed dose estimate based on a single time-point acquisition [24–26].

When camera availability is the limiting factor, multiple time-point SPECT acquisitions can be replaced with a hybrid approach that uses a combination of SPECT/CT complemented with less time-consuming yet not fully quantitative planar or whole-body imaging [27, 28]. The planar data are used for temporal sampling and do not need to be diagnostic quality, enabling further reduction in acquisition time. However, region-based determination of uptake based on 2D projections is only possible for some radiopharmaceuticals and pathologies (e.g. due to overlap of different regions of interest in anteroposterior direction). In some cases, dosimetry evaluations can also be performed without any imaging: noteworthy examples include thyroid uptake measurements or whole-body dosimetry using external radiation detectors [29]. These have the advantage that they do not impact camera availability.

Methods to reduce resource burden for verification can equally translate that required for the prescription of an activity based on a desired absorbed dose. In a theranostic setting, it is often standard practise to confirm patient eligibility with a diagnostic conjugate of the therapeutic compound. There is therefore extensive interest in using the pre-therapy images to predict therapeutic absorbed doses. This information could be used to tailor the activity prescription to deliver an optimised therapeutic absorbed dose and is an approach shown to be highly successful in SIRT [7]. Such methods have particular relevance in view of possible dose escalation beyond standard administered activity indications. Alternatively, with fractionated treatments, dosimetry performed after an initial cycle can be used to adjust the activity or number of subsequent cycles, which considerably reduces the “pre-therapy” dosimetry workload. As with level 2, the method of dosimetry does not necessarily lead to a high burden, as standard operating procedures using whole-body, blood-based and thyroid probe measurements are available for many treatments [5, 22, 24, 29, 30].

## Staff requirements

MRT dosimetry involves different competencies that must be present in a multidisciplinary team including physicians, medical physicists and technologists. Staff resourcing is a significant consideration when starting a dosimetry service. Dose calculations should be performed and completed timely prior to any concerned treatment. When scheduling times and resources, the time dedicated for data analysis and dosimetry calculations should also be considered alongside that allocated for physical measurements and scanning. The EANM survey indicated workload times required to process and analyse dosimetry data. It should be recognised that, for a new service, many of these tasks may at first take longer, while converging to improved time-efficiency as experience improves. Economy of scale will also help reducing the impact on personnel. However, commitment to resourcing and infrastructure remains usually the primary barrier to implementation of a dosimetry service.

### Role of the medical physicist

The BSS directive stipulates that a medical physics expert should act or give specialist advice, as appropriate, on matters relating to radiation physics for implementing the requirements set out in the directive. This includes taking responsibility for dosimetry, including physical measurements for the therapeutic activity to administer to the patient, estimation of absorbed doses and dose estimates to other personnel involved in the therapeutic procedures. The EANM survey demonstrated that a medical physicist was primarily involved in most aspects of the dosimetry chain but did not differentiate between the experience and the level of qualification of that medical physicist. In practical terms, many of the procedures required for dosimetry calculations can be performed by a variety of staff, including junior medical physicists, radiopharmacy lab technicians, nuclear medicine technologists, physicians or nurses. Where physics resources are scarce, it may be beneficial to explore options for shared services and cross-site collaboration. Centralising tasks such as image processing and analysis might enhance the efficiency of the dosimetry and promote the optimal use of the local resources.

### Role of the physician

The treating nuclear medicine physician having a comprehensive view of the patient situation should have appropriate training to assess and evaluate the suitability and/or the requirements for a dosimetrically optimised treatment. It should therefore be the responsibility of the physician to identify suitable patients and interpret the clinical significance of an absorbed dose, considering all patient clinical factors and other biomarkers of response and toxicity. The practitioner is responsible for the prescribed therapeutic activity and justifies the exposure to the patient, and therefore needs to be fully engaged in the multidisciplinary team responsible for performing dosimetry.

In many European centres, the nuclear medicine physician may also have a managerial role in the running of the NM department and would therefore have a clearer understanding of the resources and personnel available to commit to dosimetry. In addition to this overarching authority, the physician can play an important role in some of the practical aspects of the dosimetry regimen, such as identification and segmentation of lesions and tissues of interest. Nevertheless, to reduce the burden on the nuclear medicine physician, a multidisciplinary approach can still be adopted, whereby the initial contouring is defined by a medical physicist, a NM technologist or in a semi-automated fashion and later verified by the physician.

### Role of the nuclear medicine technologist

The role of the NM technologist should not be underestimated when developing a dosimetry service. In many countries, the NM technologist is the key person in communication with the patient, often involved in making appointments, informing them from the beginning and supporting the patient through the different dosimetry examinations. A technologist will likely spend the most time with the patient during intensive scanning regimens. Improving a patient’s experience will result in better patient cooperation and finally increase the quality of the exams. For some dosimetry procedures, the technologist may be responsible for taking samples (blood and urine) and for manipulating the samples (e.g. well counter measurements) as necessary. The NM technologist will likely also assist the medical physicist in maintaining quality assurance of devices and procedures.

It is therefore essential that NM technologists are well trained in the dosimetry protocol and feel involved and engaged in all aspects of their role. The NM technologist needs to understand the rationale for dosimetry and the requirement for accurate data collection. Good communication with the medical physicists and physicians is therefore a key factor to ensure that dosimetry remains functional and practical.

## Optimising equipment resources

Equipment is a valuable, costly and time-limited resource within a nuclear medicine department. The equipment required for dosimetry will vary depending on the specific MRT protocol, which, in many cases, can be tailored to suit equipment availability. This latter aspect is particularly sensible when first implementing dosimetry, negating or minimising the need for initial outlay costs. As the dosimetry service becomes more established, protocols can always be further developed, and additional equipment procured if necessary.

### External radiation monitors

Hand-held radiation monitors are a common piece of equipment and should be available within any nuclear medicine department. Whole-body dosimetry measurements can be made with almost any type of monitor, provided its response has been characterised. If only being used occasionally, a monitor could temporarily be brought to the patient. For regular use, it may be more appropriate to have a dedicated system configured in the treatment facility, attached to a trolley or tripod, or permanently fixed to the wall or ceiling, which can make patient positioning and measurement more efficient and reproducible. In most cases, centres opt for bespoke configurations to suit their individual needs, although commercial options, including those with direct output to PCs are available.

### Gamma counters

Due to the low activity concentrations, blood based dosimetry generally requires samples to be measured using a well-type NaI(Tl) detector (gamma counter) [22]. If a department provides a GFR service or cisternography with [^99m^Tc]-DTPA, this equipment should be readily available. For therapeutic radionuclides in general, a large flexibility exists in measuring samples at different time-points without adversely affecting other users of the gamma counter. For centres without such equipment, less costly options could be built in-house using a well-shielded sodium iodine detector or if available a high purity germanium (HPGe) detector. Radionuclide activity metres (commonly known as dose calibrators) are generally only accurate down to a few megabecquerels and are therefore insufficiently sensitive for the task. Therefore, in some cases, it is more sensible to pursue a different method of dosimetry rather than purchase this equipment for dosimetry only. The EANM provide guidelines detailing alternate methods of bone marrow dosimetry beyond that of blood sampling [22].

### Thyroid uptake probes

Thyroid uptake probes consist of a thallium-activated sodium iodide crystal coupled to a multiscale analyser or energy discriminator and counting system. The probe is collimated with lead to give a field of view appropriate to cover the patient neck area. Various dedicated commercial options exist, or a system could potentially be constructed in-house if an appropriate detector is available. For centres without a dedicated probe, gamma camera imaging may also be performed to provide the same information. The need for a dedicated system is then a trade-off between purchase cost and gamma camera capacity. While these systems are primarily designed to measure uptake of I-123 or I-131 in the thyroid, they can also be used for other measurements such as whole-body count rates or activity in blood samples. For such alternative uses, the probe response would first need to be characterised to avoid dead-time effects. In such cases, measurement of high-activity blood samples can be delayed until sufficiently decayed.

### Imaging equipment

Most forms of image-based dosimetry are currently performed using gamma cameras or SPECT systems. While SPECT/CT imaging is often recommended, it is, for several applications, also possible to use methods based on SPECT only or planar gamma camera imaging [24, 28]. It is evident that a centre providing a theranostic service needs access to at least one gamma camera.

In general, patient scanning may require up to 2 h of camera time for individual patients when multiple imaging sessions are performed [19]. However, reducing the number of time-points appears feasible for some treatments, when the pharmacokinetics are well described. Single time-point protocols have been suggested for both [^177^Lu]-DOTATATE kidney dosimetry and [^177^Lu]-PSMA-617 [31, 32]. In the future, acquisition times could be reduced through technological advancements such as the introduction of AI-based reconstruction protocols and acceleration of SPECT/CT acquisition protocols [33].

Specific MRT applications exist for PET/CT used to directly image [^90^Y]-microspheres for post therapy dosimetry verification after radioembolisation and some β^+^ emitter diagnostic companions included in the theragnostic workflow. Alternatives exist for centres without PET scanners, in the form of the gamma camera–based bremsstrahlung imaging for ^90^Y [34] or single photon emission–based tracers such as ^111^In or ^99m^Tc in place of ^68^ Ga [35]. It is worth noting that Bremsstrahlung imaging of ^90^Y is typically a non-quantitative procedure and less suited for accurate dosimetry, but instead useful for qualitative treatment verification [34]. While anatomical information is readily obtainable through the CT component of hybrid scanners, extraction of volume measures or co-registration of images from, e.g. stand-alone CTs are also possible and a lack of CT should not be a barrier for a centre wishing to perform dosimetry.

### Software

When considering the entire dosimetry workflow, the image post-processing aspects specifically required for the dose calculation typically takes up two thirds of the total personnel resources time. For this reason, the selection and implementation of software used for dosimetry is of great importance. Due to the absence of commercial dosimetry software in the past, dosimetry calculations have long been relying on in-house solutions. However, an increasing number of commercial dosimetry software solutions have become available over the last years. Most are both CE marked and/or FDA-approved [36, 37], but very heterogeneous in function and application. The cost of commercial dosimetry packages may require a large number of patients and reimbursement to be cost-effective, and affordable to a department. Academic and freeware software may therefore be an alternative option. For less advanced calculations, it is often reasonable to employ basic image computing platforms for viewing and segmentation, in combination with spreadsheets or freely available general-purpose programming languages [38, 39]. The personnel effort required to implement an academic or freeware-based solution is likely to be greater than for a commercial software solution. However, the former offers more flexibility and allows the users to develop a bespoke solution tailored to the individual centre. Provided sufficient skills and knowledge of the user/developer are available. An adequate internal benchmarking/validation system should be developed.

## Discussion

The field of MRT is rapidly evolving and expanding its clinical prominence to multiple new tumour entities [40]. The approval of 177Lu-PSMA 617 by FDA and EMA manifests the successful expansion of MRT to a high-volume indication such as metastatic castration–resistant prostate cancer. The pivotal trial (VISION) leading to approval used a standard activity (7.4 GBq) in four and up to six cycles of 177Lu-PSMA 617 confirming a median overall survival and median progression-free survival benefit of 4.0 and 5.3 months compared to the SOC group [41]. However, more than 50% of patients in the treatment arm did not achieve a PSA decrease of > 50%. In consideration of an overall well tolerated one size fits all dosing approach, it can be discussed if a more personalised approach taking advantage of a large therapeutic window might increase the rate of responders. The currently ongoing read out of the VISION dosimetry sub-study will provide information how the therapeutic activities can be individually escalated when based on normal organ doses as well as provide intel on achievable (and required) tumour doses. An improvement in response and more importantly survival would clearly justify the added effort, cost and exposure of dosimetry to patients, medical experts and society.

In the wake of an ever-increasing number of new MRT programmes and a better understanding of radiobiology [42], dosimetry has the opportunity especially in early phases of clinical development to fast-track clinical translation, improve the understanding of a potential therapeutic index and reduce the risk of late phase clinical trial failures.

## Conclusions

Dosimetry plays a key role in the personalisation and continued optimisation of theragnostic nuclear medicine. Procedures to implement dosimetry can be optimised to suit the needs and resources of the department.

## Supplementary Information

Below is the link to the electronic supplementary material.

Supplementary file1 (DOCX 69 KB)

## Declarations

### Liability

This guide summarises the views of the co-authoring EANM Committee members. It reflects recommendations for which the EANM cannot be held responsible. The recommendations should be taken into the context of good practise of nuclear medicine and do not substitute for national and international legal or regulatory provisions.
