# Correction to: EANM enabling guide: how to improve the accessibility of clinical dosimetry

**DOI:** 10.1007/s00259-023-06304-2

**Published:** 2023-06-29

**Authors:** Jonathan Gear, Caroline Stokke, Christelle Terwinghe, Silvano Gnesin, Mattias Sandström, Johannes Tran‑Gia, Marta Cremonesi, Francesco Cicone, Fredrik Verburg, Roland Hustinx, Luca Giovanella, Ken Herrmann, Pablo Minguez Gabiña

**Affiliations:** 1 Joint Department of Physics, Royal Marsden NHSFT & Institute of Cancer Research, Sutton, UK; 2 Division of Radiology and Nuclear Medicine, Oslo University Hospital, Oslo, Norway; 3 Department of Physics, University of Oslo, Oslo, Norway; 4 Department of Nuclear Medicine, Universitair Ziekenhuis Leuven, Louvain, Belgium; 5 Institute of Radiation Physics, Lausanne University Hospital, University of Lausanne, Lausanne, Switzerland; 6 Section of Nuclear Medicine and PET, Department of Surgical Sciences, Uppsala University, Uppsala, Sweden; 7 Sweden & Section of Medical Physics, Department of Immunology, Genetics and Pathology, Uppsala University, 751 85 Uppsala, Sweden; 8 Department of Nuclear Medicine, University Hospital Würzburg, Oberdürrbacher Str. 6, 97080 Würzburg, Germany; 9 Radiation Research Unit, Department of Medical Imaging and Radiation Sciences, Istituto Europeo Di Oncologia, IRCCS, Milan, Italy; 10 Department of Experimental and Clinical Medicine, Neuroscience Research Centre, PET/RM Unit, “Magna Graecia” University of Catanzaro, Catanzaro, Italy; 11 Nuclear Medicine Unit, University Hospital “Mater Domini, Catanzaro, Italy; 12 Department of Radiology and Nuclear Medicine, Erasmus Medical Center, Rotterdam, the Netherlands; 13 Division of Nuclear Medicine and Oncological Imaging, University Hospital of Liège, Liège, Belgium; 14 GIGA‑CRC in Vivo Imaging, University of Liège, Liège, Belgium; 15 Clinic for Nuclear Medicine and Molecular Imaging, Imaging Institute of Southern Switzerland, Ente Ospedaliero Cantonale, Bellinzona, Switzerland; 16 Department of Nuclear Medicine, University of Duisburg-Essen, Duisburg, Germany; 17 German Cancer Consortium (DKTK)-University Hospital Essen, Essen, Germany; 18 Department of Medical Physics and Radiation Protection, Gurutzeta-Cruces University Hospital/Biocruces Health Research Institute, Barakaldo, Spain; 19 Department of Applied Physics, Faculty of Engineering, UPV/EHU, Bilbao, Spain

**Correction to: European Journal of Nuclear Medicine and Molecular Imaging (2023) 50:1861–1868**

**https://doi.org/10.1007/s00259-023-06226-z**

The article EANM enabling guide: how to improve the accessibility of clinical dosimetry, written by Jonathan Gear, Caroline Stokke, Christelle Terwinghe, Silvano Gnesin, Mattias Sandström, Johannes Tran‑Gia, Marta Cremonesi, Francesco Cicone, Fredrik Verburg, Roland Hustinx, Luca Giovanella, Ken Herrmann, and Pablo Minguez Gabiña, was originally published Online First without Open Access. After publication in volume 50, issue 7, page 1861–1868, the author decided to opt for Open Choice and to make the article an Open Access publication. Therefore, the copyright of the article has been changed to © The Authors 2023 and the article is forthwith distributed under the terms of the Creative Commons Attribution 4.0 International License, which permits use, sharing, adaptation, distribution and reproduction in any medium or format, as long as you give appropriate credit to the original author(s) and the source, provide a link to the Creative Commons licence, and indicate if changes were made.

The original article has been corrected.

