# Supplementary material for: EANM enabling guide: how to improve the accessibility of clinical dosimetry
Source: Eur J Nucl Med Mol Imaging. 2023 Apr 22;50(7):1861–8. doi: 10.1007/s00259-023-06226-z (PMC10287783; doi:10.1007/s00259-023-06226-z)
Supplement: Supplementary file 1 — Supplementary file1 (DOCX 69 KB) [file 259_2023_6226_MOESM1_ESM.docx]

# Clinical Indications & Examples:

# SUPPLIMENTARY MATERIAL TO THE EANM ENABLING GUIDE: HOW TO IIMPROVE THE ACCESSIBILITY OF CLINICAL DOSIMETRY

In the following a brief overview is given of some of the different approaches to dosimetry for common nuclear medicine therapies. The rationale for dosimetry is presented alongside two possible, but different dosimetry strategies. The advantages and disadvantages of each method are summarised. These methodologies have been highlighted to demonstrate the vast differences in methods and resources that could be applied. These methodologies are neither exhaustive nor exclusive and an alternative or combination of each technique could also be applied.

## I-131 Sodium Iodide

The rationale behind dosimetry prior to therapy is to determine the ^131^I activity that is most likely to lead to therapeutic success whilst limiting the radiation exposure to the amount necessary [1]. A prospective randomized study on radioiodine treatment in patients with Graves’ disease showed a similar outcome in patients treated with 555 MBq and patients who received a target absorbed doses of 100 Gy, but also that those patients treated with 555 MBq who received absorbed doses higher than 200 Gy had a higher success rate [2]. Also, a retrospective study has shown that by delivering dosimetry-driven treatments, the activity to achieve the desired response can be reduced [3]. However, patients with cardiovascular risk factors may benefit from a definitive treatment with a fixed higher administered activity to ensure hypothyroidism is rapidly achieved [4]. The prospective randomized comparison in Graves disease (or any other benign thyroid disease) using standard activities vs. personalized activities attempting to achieve 300-400 Gy has not been performed yet, hence lacking the confirmation of superiority of one another

EANM guidelines recommend that absorbed doses of 300–400 Gy should be used to ablate autonomous nodules, and in patients with Graves’ disease, 200–300 Gy [1], supported by a systematic review study by Taprogge et al [5]. To deliver a prescribed absorbed dose requires a pre-therapy dosimetry study (level 3). Such a study should not be associated with excessive effort, for neither the nuclear medicine department nor the patient. EANM standard operating procedures are available to aid centres in designing and implementing such a study, with sufficient scope for the centre to adjust as appropriate to the resources available [6]. Table 1 highlights two possible study methodologies using I-131 that could be implemented, although I-123 could also be used as an alternative. These methods are not an exhaustive list and aspects o f each approach could equally be taken to form an alterative regimen.

| Clinical Indication | Benign thyroid disease without cardiovascular risk factors |
| --- | --- |
| Level of Dosimetry | EANM level 3 (prescription to absorbed dose) |
|  | |
| APPROACH A | APPROACH B |
| Methodological Description | |
| - Thyroid pertechnetate uptake study - Target volume determined by ultrasound - Tracer administration of 10 MBq of I-131 - Thyroid uptake scintigraphy at 4 hours p.i. - Thyroid uptake scintigraphy at 24 hours p.i. - Thyroid uptake scintigraphy at 72 hours p.i. - Thyroid uptake scintigraphy at 144 hours p.i. - Absorbed dose calculation - Therapeutic administration of I-131 | - Target volume determined from pertechnetate uptake study - Tracer administration of 2 MBq of I-131 - Thyroid uptake probe measurement at 5 – 8 days P.I. - Absorbed dose calculation - Therapeutic administration of I-131 |
| Advantages | |
| - Ultrasound scan gives accurate mass estimate - Calculation of patent specific half-life reduces uncertainty (<10%) in dose calculation. - Multi-time point uptake allows uncertainty in absorbed dose to be determined. - Gamma camera quantification is more accurate | - If pertechnetate scan is standard of care, use for mass estimate negates the need for additional ultrasound scan. - Single time point method reduces number of hospital visits - Use of thyroid uptake probe does not require use of other NM resources |
| Disadvantages | |
| - Additional ultrasound scan needed - Extra hospital visits and measurements needed. - High activity required for gamma camera measurements - Gamma camera time may be limited | - Large margin of error using scintigraphy for thyroid mass estimate - Errors exceeding a factor of two are possible in individual patients if the uptake is measured after 1 day. The potential for error is slightly lower for uptake assessments after 2 days - Gamma probe is not standard equipment in every centre. |

Table 1: Example dosimetry schemas for I-131 therapy of benign thyroid disease.

## Lu-177 DOTATATE

The joint IAEA, EANM, and SNMMI practical guidance on peptide receptor radionuclide therapy indicates that patient-specific dosimetry can provide valuable information to assess organ-specific radiation absorbed doses and to assess the risk of delayed kidney toxicity, particularly in patients with known risk factors, including longstanding and poorly controlled hypertension and diabetes mellitus [7] . The EANM dosimetry recommendations for dosimetry of ^177^Lu-labelled somatostatin-receptor targeting ligands [8] provides comprehensive guidance and information for clinical implementation. There are numerous methods and approaches for Lu-177 dosimetry, each with advantages and disadvantages. Table 2 provides just two example regimens that could be considered for dosimetry assessment in cases where there is particular concern of kidney toxicity. In this example, an absorbed dose limit of 23 Gy over 4 cycles has been suggested. However, whilst extrapolated from external beam-radiotherapy [9], this value is not a confirmed toxicity threshold, for MRT as the dose rates and micro-distribution of the radiopharmaceutical can be more heterogeneous than external beam. Lower toxicity incidents have hence been observed at significantly higher doses in some retrospective studies [10-12].

| Clinical Indication | Expression of sstr2, or metastatic or inoperable neuroendocrine tumours with poor kidney function |
| --- | --- |
| Level of Dosimetry | EANM level 3 (prescribe to a dose constraint) |
|  | |
| APPROACH A | APPROACH B |
| Methodological Description | |
| - 7400 MBq Lu-177 administered for cycle 1. - SPECT/CT imaging of kidneys and lesions at 24 hours p.i. - SPECT/CT imaging of kidneys and lesions at 96 hours p.i. - SPECT/CT imaging of kidneys and lesions at 168 hours p.i. - Organ/lesion delineation on CT - Absorbed dose calculation for kidneys and lesions   Provided AD_kidney_ for the four cycles will be less than 23 Gy then administer next cycle and repeat | - 7400 MBq Lu-177 administered for cycle 1 - SPECT/CT imaging of kidneys at 96 hours and use a population elimination (52) hours for kidneys - Kidneys delineation on SPECT or CT - Absorbed Dose calculation of kidneys   Ensure AD_kidney_ x4 <23 Gy  Administer next 3 cycles with SPECT/CT imaging of kidneys at 96 hours p.i. |
| Advantages | |
| - Highly accurate dose calculation using multiple SPECT/CT - Multi-time point scans allow uncertainty in absorbed dose to be expressed. - Risk of toxicity is limited - Probability for response is indicated by lesion absorbed doses - Prediction of absorbed dose is verified at all cycles | - Fairly accurate dose calculation - Risk of toxicity is reduced ensuring kidney doses are below a toxicity threshold - Low scanning burden for patient and department |
| Disadvantages | |
| - SPECT/CT is time consuming and gamma camera time may be limited - Protocol demands up to additional 12 low-dose CT exposures - Depending on the duration of the hospitalization, several additional hospital visits may be required for the additional scans. - Treatment administration not maximised, just kept below the 23 Gy constraint | - One timepoint approach is less accurate - Lesion doses are generally not calculated so efficacy is uncertain - Biokinetics of kidney unknown. - Patients with renal impairment may not follow the assumed population biokinetics - Treatment administration not maximised, just kept below the 23 Gy constraint |

Table 2. Example dosimetry schemas for Lu-177 DOTATATE for neuroendocrine tumours.

## I-131 mIBG

I-131 mIBG is used as a radiotherapeutic metabolic agent in neuroectodermal tumours of the sympathetic nervous system with prevalent use in treating paediatric neuroblastoma. The EANM procedure guidelines for 131-I mIBG therapy indicate that the organ that limits the activity to be administered is predominantly the red marrow [13]. The EANM Dosimetry Committee series on standard operational procedures for internal dosimetry for 131-I mIBG treatment of neuroendocrine tumours [14] suggests using whole-body dosimetry as a surrogate for red marrow dosimetry. Both the EANM and ICRU strongly recommend dosimetrically optimised activity prescriptions for paediatric administrations [15, 16], and as such some centres and clinical trials now opt to treat to a target 4 Gy whole-body dose, usually delivered over 2 cycles [17-20]. The activity administered for the first cycle is determined from patient weight and subsequent whole-body dosimetry used to determine the activity of the second infusion. Table 3 presents two dosimetry regimens which both set out to deliver a dosimetrically optimised therapy. As with previous examples, one of these approaches requires less resources and the pro’s and con’s of each are briefly outlined.

| Clinical Indication | Patients with metastatic neuroblastoma with a poor response to Induction Chemotherapy |
| --- | --- |
| Level of Dosimetry | EANM level 3 (prescription to absorbed dose) |
|  | |
| APPROACH A | APPROACH B |
| Methodological Description | |
| - 444 MBq per kg I-131 administered for cycle 1. - WB counting using ceiling mounted detector 4 times per day until patient activity <300 MBq - SPECT/CT imaging of lesions at 24 hours p.i. - SPECT/CT imaging of lesions at 72 hours p.i. - SPECT/CT imaging of lesions at 120 hours p.i. - Lesions delineation on CT - Absorbed dose calculation of whole body and lesions - Administer 2^nd^ cycle to deliver AD_WB_ = 4 Gy and repeat dosimetry. | - 444 MBq per kg I-131 administered for cycle 1. - WB counting performed once per day using dose rate monitor until patient activity <300 MBq - Qualitative image at 72 hours to verify treatment delivery. - Absorbed dose calculation to whole body - Administer 2^nd^ cycle to deliver AD_WB_ = 4 Gy |
| Advantages | |
| - WB measurement system can be used by all staff groups and patient’s parents, - Highly accurate dose calculation using multiple SPECT/CT - All scans & measurements occur whilst patient is in hospital - Multi-time points allow uncertainty in absorbed dose to be expressed. - Treatment efficacy is verified by determining lesion doses. | - Dose rate meter readily available in NM department. - All measurements occur whilst patient is in hospital - Multi-time points allow uncertainty in absorbed dose to be expressed. - Qualitative images can be used to ensure distribution of uptake is as expected |
| Disadvantages | |
| - WB measurement system is bespoke and requires installation. - SPECT/CT is time consuming and gamma camera may be time limited - Potential radiation exposure to scanning staff - Protocol demands up to 6 additional low-dose CT exposures | - Dose rate measurements are less frequent - Potential radiation exposure to personnel taking dose rate measurements - Lesion doses are not calculated so efficacy is not verified - WB dose is not measured on 2^nd^ cycle so actual AD_WB_ is unknown |

Table 3. Example dosimetry schemas for I-131 mIBG therapy of metastatic neuroblastoma.

## Y-90 Radioembolization

The selective loco-regional permanent implantation of ^90^Y loaded microspheres is a well-established therapeutic option for the treatment of the primary hepatic carcinoma and metastasis. This technique also known as selective internal radiation therapy (SIRT), transarterial radioembolisation (TARE) or ^90^Y hepatic radioembolisation, demonstrated the key role of dosimetry in improving patient outcome [21, 22]. Based on clinical results, present international recommendations for SIRT considers both predictive and post-therapy absorbed dose assessment [23-25], hence in compliance with the European BSS. The determination of the patient specific ^90^Y therapeutic activity administration relies on predictive dosimetry calculations achieved in a treatment simulation of the therapeutic ^90^Y activity deposition by the transarterial hepatic administration of a diagnostic activity of ^99m^Tc macro-aggregate albumins (^99m^Tc-MAA) imaged in SPECT/CT.

For predictive dosimetry, planar imaging is sufficient for extrahepatic lung and gastro-intestinal shunt assessments, but SPECT/CT has superior spatial localisation and quantification abilities and can be used for determination of uptake in lesions and normal liver.

Qualitative post-treatment verification of the appropriate deposition of ^90^Y-labelled microspheres is performed with bremsstrahlung planar and SPECT/CT imaging. This step is essential for assessing possible post-therapy extrahepatic shunt and enable appropriate and timely medication if needed. Alternatively. a post-treatment absorbed dose verification in lesions and hepatic non-tumour tissues can be obtained from quantitative ^90^Y PET/CT (typically 15-30 minutes in single bed position centred on the liver), depending on the PET system available..

The assumption of permanent local deposition of the ^90^Y-loaded microspheres reduces the need of quantitative imaging to only a single acquisition. Typically, ^99m^Tc-MAA SPECT/CT is acquired promptly within an hour after the administration, while ^90^Y PET/CT is acquired within a few hours post-therapeutic implantation before patient discharge from the hospital.

| Clinical Indication | Patients with unresectable hepatic carcinoma or liver metastases. |
| --- | --- |
| Level of Dosimetry | EANM level 3 (prescribe to dose with post-treatment absorbed dose verification. |
|  | |
| APPROACH A | APPROACH B |
| Methodological Description | |
| - Diagnostic administration ^99m^Tc-MAA. - SPECT/CT imaging of the abdomen (liver and gastro-intestinal tract) within 1h p.i. - Planar or SPECT/CT imaging for lung shunt assessment, within 1h p.i. - Liver tumour and non-tumour delineation on CT, lungs on CT or planar emission imaging. - Voxel dosimetry (Mean absorbed dose and DVH) for tumour and non-tumour hepatic volumes and lungs (if lung shunt >0) - Administer activity based on voxel dosimetry considering DVH information and mean dose threshold for efficacy (tumour) and safety (non-tumour liver) - Post-treatment dosimetry based on ^90^Y PET/CT within few hours post-administration. | - Diagnostic administration ^99m^Tc-MAA. - SPECT/CT imaging of the abdomen (liver and gastro-intestinal tract) within 1h p.i. - Planar or SPECT/CT imaging for lung shunt assessment, within 1h p.i. - Liver tumour and non-tumour delineation on CT, lungs on CT or planar emission imaging. - Mean absorbed dose calculations for tumour and non-tumour hepatic volumes and lungs (if lung shunt >0). - Administer activity based on partition model considering mean dose threshold for efficacy (tumour) and safety (non-tumour liver) |
| Advantages | |
| - Improved treatment personalization and expected efficacy taking into account the spatial (intra- and inter-lesion) heterogeneity of absorbed dose distribution. - Post-therapy dosimetry verification allows for better tailoring future therapy sessions and optimal patient management. - Post-therapy dosimetry provide valuable information for dose-effects studies. - Risk of toxicity is limited | - Reasonably accurate predictive dosimetry (mean doses in the tumour and non-tumour compartments) based on the partition model dosimetry - Lower scanning burden for patient and department - Risk of toxicity is limited - No need for a specific dosimetry software, an electronic spreadsheet can suffice. |
| Disadvantages | |
| - Typically requires specific software implementing 3D voxel dosimetry - Not demonstrated clinical superiority of voxel dosimetry over partition model dosimetry. - Extra time and resources required for post-SIRT Y-90 Dosimetry verification. | - Assumption of close agreement between the predicted and the actual therapeutic absorbed dose distribution. Not always true [26, 27]. - Neglect possible absorbed dose heterogeneity in targeted lesion and non-tumour parenchyma. - No post-treatment absorbed dose verification. |

Table 4. Example dosimetry schemas for Y-90 microsphere treatment of hepatic carcinoma.

## References

1. Stokkel, M.P.M., et al., *EANM procedure guidelines for therapy of benign thyroid disease.* European Journal of Nuclear Medicine and Molecular Imaging, 2010. **37**(11): p. 2218-2228.

2. Peters, H., et al., *Treatment of Graves' hyperthyroidism with radioiodine: Results of a prospective randomized study.* Thyroid, 1997. **7**(2): p. 247-251.

3. Hyer, S.L., et al., *Dosimetry-based treatment for Graves' disease.* Nuclear Medicine Communications, 2018. **39**(6): p. 486-492.

4. Okosieme, O.E., et al., *Primary therapy of Graves' disease and cardiovascular morbidity and mortality: a linked-record cohort study.* Lancet Diabetes & Endocrinology, 2019. **7**(4): p. 278-287.

5. Taprogge, J., et al., *A Systematic Review and Meta-Analysis of the Relationship Between the Radiation Absorbed Dose to the Thyroid and Response in Patients Treated with Radioiodine for Graves' Disease.* Thyroid, 2021. **31**(12): p. 1829-1838.

6. Lassmann, M., et al., *EANM Dosimetry Committee series on standard operational rocedures for pre-therapeutic dosimetry I: blood and bone marrow dosimetry in differentiated thyroid cancer therapy.* European Journal of Nuclear Medicine and Molecular Imaging, 2008. **35**(7): p. 1405-1412.

7. Zaknun, J.J., et al., *The joint IAEA, EANM, and SNMMI practical guidance on peptide receptor radionuclide therapy (PRRNT) in neuroendocrine tumours.* European Journal of Nuclear Medicine and Molecular Imaging, 2013. **40**(5): p. 800-816.

8. Gleisner, K.S., et al., *EANM dosimetry committee recommendations for dosimetry of 177Lu-labelled somatostatin-receptor- and PSMA-targeting ligands.* European Journal of Nuclear Medicine and Molecular Imaging, 2022. **49**(6): p. 1778-1809.

9. Emami, B., et al., *Tolerance of Normal Tissue to Therapeutic Irradiation.* International Journal of Radiation Oncology Biology Physics, 1991. **21**(1): p. 109-122.

10. Bodei, L., et al., *Receptor radionuclide therapy with Y-90-[DOTA](0)-Tyr(3)-octreotide (Y-90-DOTATOC) in neuroendocrine tumours.* European Journal of Nuclear Medicine and Molecular Imaging, 2004. **31**(7): p. 1038-1046.

11. Bodei, L., et al., *Long-term evaluation of renal toxicity after peptide receptor radionuclide therapy with Y-90-DOTATOC and Lu-177-DOTATATE: the role of associated risk factors.* European Journal of Nuclear Medicine and Molecular Imaging, 2008. **35**(10): p. 1847-1856.

12. Bodei, L., et al., *Long-term tolerability of PRRT in 807 patients with neuroendocrine tumours: the value and limitations of clinical factors.* European Journal of Nuclear Medicine and Molecular Imaging, 2015. **42**(1): p. 5-19.

13. Giammarile, F., et al., *EANM procedure guidelines for I-131-meta-iodobenzylguanidine (I-131-mIBG) therapy.* European Journal of Nuclear Medicine and Molecular Imaging, 2008. **35**(5): p. 1039-1047.

14. Gear, J., et al., *EANM Dosimetry Committee series on standard operational procedures for internal dosimetry for I-131 mIBG treatment of neuroendocrine tumours.* Ejnmmi Physics, 2020. **7**(1).

15. Konijnenberg, M., et al., *EANM position paper on article 56 of the Council Directive 2013/59/Euratom (basic safety standards) for nuclear medicine therapy.* Eur J Nucl Med Mol Imaging, 2021. **48**(1): p. 67-72.

16. Sgouros, G., et al., *ICRU Report 96, Dosimetry-Guided Radiopharmaceutical Therapy*, in *Journal of the ICRU*. 2022, THE INTERNATIONAL COMMISSION ON RADIATION UNITS AND MEASUREMENTS.

17. Buckley, S.E., et al., *Whole-Body Dosimetry for Individualized Treatment Planning of I-131-MIBG Radionuclide Therapy for Neuroblastoma.* Journal of Nuclear Medicine, 2009. **50**(9): p. 1518-1524.

18. Gaze, M.N., et al., *Feasibility of dosimetry-based high-dose (131)I-meta-iodobenzylguanidine with topotecan as a radiosensitizer in children with metastatic neuroblastoma.* Cancer Biotherapy and Radiopharmaceuticals, 2005. **20**(2): p. 195-199.

19. Genolla, J., et al., *Dosimetry-based high-activity therapy with I-131-metaiodobenzylguanidine (I-131-mIBG) and topotecan for the treatment of high-risk refractory neuroblastoma.* European Journal of Nuclear Medicine and Molecular Imaging, 2019. **46**(7): p. 1567-1575.

20. Rubio, P.M., et al., *MIBG Therapy for Neuroblastoma: Precision Achieved With Dosimetry, and Concern for False Responders.* Frontiers in Medicine, 2020. **7**.

21. Garin, E., et al., *Major impact of personalized dosimetry using 90Y loaded glass microspheres SIRT in HCC: Final overall survival analysis of a multicenter randomized phase II study (DOSISPHERE-01).* Journal of Clinical Oncology, 2020. **38**(4).

22. Levillain, H., et al., *Personalised radioembolization improves outcomes in refractory intra-hepatic cholangiocarcinoma: a multicenter study.* European Journal of Nuclear Medicine and Molecular Imaging, 2019. **46**(11): p. 2270-2279.

23. Levillain, H., et al., *International recommendations for personalised selective internal radiation therapy of primary and metastatic liver diseases with yttrium-90 resin microspheres.* European Journal of Nuclear Medicine and Molecular Imaging, 2021. **48**(5): p. 1570-1584.

24. Salem, R., et al., *Clinical and dosimetric considerations for Y90: recommendations from an international multidisciplinary working group.* European Journal of Nuclear Medicine and Molecular Imaging, 2019. **46**(8): p. 1695-1704.

25. Weber, M., et al., *EANM procedure guideline for the treatment of liver cancer and liver metastases with intra-arterial radioactive compounds.* European Journal of Nuclear Medicine and Molecular Imaging, 2022. **49**(5): p. 1682-1699.

26. Gnesin, S., et al., *Partition Model Based Tc-99m-MAA SPECT/CT Predictive Dosimetry Compared with Y-90 TOF PET/CT Posttreatment Dosimetry in Radioembolization of Hepatocellular Carcinoma: A Quantitative Agreement Comparison.* Journal of Nuclear Medicine, 2016. **57**(11): p. 1672-1678.

27. Richetta, E., et al., *PET-CT post therapy dosimetry in radioembolization with resin Y-90 microspheres: Comparison with pre-treatment SPECT-CT Tc-99m-MAA results.* Physica Medica-European Journal of Medical Physics, 2019. **64**: p. 16-23.
